# Whole genomes reveal subpopulations and isolation-by-distance patterns in the Norwegian lemming

**DOI:** 10.1186/s12915-026-02568-w

**Published:** 2026-03-06

**Authors:** Isabelle Sofie Feinauer, Francesco Ravasini, Vendela Kempe Lagerholm, Johannes Måsviken, Remi-Andre Olsen, Lucile Soler, Estelle Proux-Wera, Ignas Bunikis, Henrik Lantz, Kerstin Lindblad-Toh, Dorothee Ehrich, Rolf A. Ims, Heikki Henttonen, Nina E. Eide, Øystein Flagstad, Karin Norén, Anders Angerbjörn, Love Dalén

**Affiliations:** 1 Centre for Palaeogenetics, Stockholm, Sweden; 2 Department of Zoology, Stockholm University, Stockholm, Sweden; 3 Department of Bioinformatics and Genetics, Swedish Museum of Natural History, Stockholm, Sweden; 4 Sapienza University of Rome, Rome, Italy; 5 Department of Archaeology and Classical Studies, Stockholm University, Stockholm, Sweden; 6 Science for Life Laboratory, Department of Biochemistry and Biophysics, Stockholm University, Stockholm, Sweden; 7 Science for Life Laboratory, National Bioinformatics Infrastructure Sweden (NBIS), Uppsala University, Uppsala, Sweden; 8 Department of Medical Biochemistry and Microbiology, Uppsala University, Uppsala, Sweden; 9 Department for Immunology, Genetics and Pathology, Uppsala University, Uppsala, Sweden; 10 Science for Life Laboratory, Uppsala University, Uppsala, Sweden; 11 Broad Institute of MIT and Harvard, Cambridge, MA USA; 12 Department of Arctic and Marine Biology, Faculty of Biosciences, Fisheries and Economics, UiT, The Arctic University of Norway, Tromsø, Norway; 13 Natural Resources Institute Finland, Helsinki, Finland; 14 Norwegian Institute of Nature Research, Trondheim, Norway

**Keywords:** Norwegian lemming, Whole genomes, Population structure, Population genomics, Isolation by distance, Fennoscandia

## Abstract

**Background:**

The Norwegian lemming (*Lemmus lemmus*) is a small rodent endemic to the Fennoscandian alpine and arctic tundra. The species is known for cyclic population outbreaks and mass movements during peak years. Previous research based on microsatellites revealed high genetic variation but a weak population structure in the Norwegian lemming.

**Results:**

In this study, we revisit the population structure of the species using genome-wide data. To do this, we generated a high-quality de novo reference genome for *Lemmus lemmus*, and resequenced genomes to 2.5–5 × coverage, from 86 lemmings sampled across the species’ entire geographic distribution. Our results reveal that the population is geographically structured into distinct subpopulations, with an overall pattern characterised by isolation-by-distance among subpopulations. Furthermore, our results are consistent with earlier work suggesting that the species survived the last ice age within a northern refugium.

**Conclusions:**

Together, these findings provide a genome-wide perspective on today’s population structure of the Norwegian lemming. In addition, we provide a de novo reference genome, which we believe will be a valuable resource to the research community.

**Supplementary Information:**

The online version contains supplementary material available at 10.1186/s12915-026-02568-w.

## Background

The Norwegian lemming (*Lemmus lemmus*;* L. lemmus*; Linnaeus 1758) is an iconic small rodent, and the only mammal endemic to Fennoscandia [1]. It is recognised for its aggressive behaviour and striking yellow-black coat coloration, which may constitute aposematic traits [2]. The Norwegian lemming is known to undergo cyclic population dynamics, with dramatic population outbreaks and subsequent crashes every 3–5 or more years [3]. Moreover, Norwegian lemmings are known for mass movements, and can migrate over distances more than 100 km [4]. It has been proposed that the repeated population crashes result in reduced genetic diversity and marked population structure [5]. Surprisingly, earlier work based on microsatellite data has indicated the opposite, and found a high genetic variation and surprisingly weak population structure in the species [6]. These results support the hypothesis that the high dispersal rate of Norwegian lemmings could potentially lead to connectivity of subregions, with gene flow counteracting a clear genetic structuring [7]. However, these results were obtained from analysing only twelve microsatellite loci, and the inferences based on these may thus have had limited statistical power.

The Norwegian lemming has likely evolved during the Late Pleistocene and has one of the youngest speciation times among mammals [8–11]. A recent study analysing the phylogenomic relationship of species of the genus *Lemmus* [11] suggested that the Norwegian lemming and its sister species, the Siberian lemming (*Lemmus sibiricus*), indeed only diverged around ~ 36 thousand years ago, just before the Last Glacial Maximum (LGM; 26.5 to 19 kya [12]). During the Last Glacial Maximum, Fennoscandia was covered by the Fennoscandian Ice Sheet [13]. Many species survived the LGM by retracting into southern refugia, from where they subsequently recolonised Fennoscandia after the ice sheet retreated [14–16]. Interestingly, based on short mitochondrial markers, a different scenario has been proposed for the Norwegian lemming [9, 10], in which the species survived the LGM locally in an ice-free northern refugium and expanded across Fennoscandia at the end of the last glaciation. A possible location for such a local refugium is Andøya [10, 17].

**Fig. 1:**
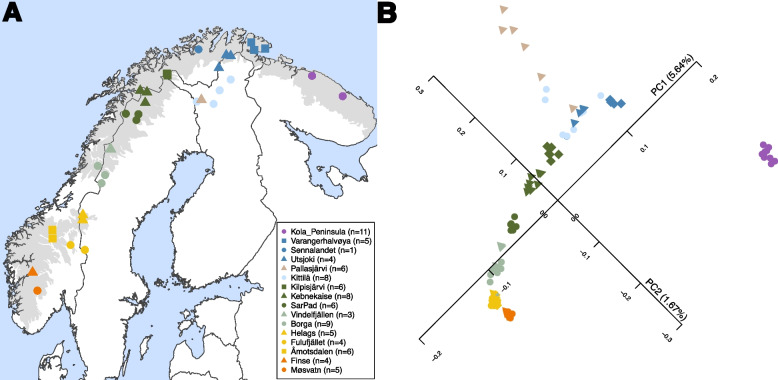
**A** Sampling locations of the modern Norwegian lemming (*L. lemmus*) samples. The plot was generated in R v4.3.2 (2023–10–31) [18], using the rworldmap v1.3–8 [19] and the sf v1.0–19 packages [20, 21]. Grey shaded areas show tundra regions, generated using the WWF shapefile “Terrestrial Ecoregions of the World (TEOW)”, biome 11 [22]. Colours and symbols correspond to sampling location, as indicated in the legend, with number of samples per location given in parentheses. **B** Principal Component Analysis of the modern *L. lemmus* samples, with PC1 displayed on the *x*-axis and PC2 on the *y*-axis. Percentages in parentheses show the variance explained

So far, genomic research on the Norwegian lemming has focussed on the phylogenetic relationships between *Lemmus* species and detecting gene flow among the two sister species *L. lemmus* and *L. sibiricus* [11]. In this study, we present a high-quality de novo reference genome for the Norwegian lemming, along with 86 newly generated population genomes resequenced to 2.5–5 × coverage, sampled across the species’ entire geographic range in Fennoscandia (Fig. 1A; Additional file 1: Table S1). This extensive dataset of high-quality genomes allows us to revisit the modern-day population structure of the Norwegian lemming, a research question that has so far only been addressed using short mitochondrial markers and microsatellites.

The aim of this study was to assess how the Norwegian lemming’s evolutionary history and migratory behaviour have influenced its population genomic structure. First, although the species’ high migration rate may have resulted in weak genetic structure, primarily reflected in a gradient of allele frequencies across Fennoscandia [6], the alpine and arctic tundra is not a continuous habitat as forested valleys and fjords interrupt it in several areas. We therefore aimed to test whether whole-genome data could reveal geographically distinct subpopulations across the Norwegian lemming’s range. Second, based on the earlier hypothesis of a postglacial expansion from an LGM northern refugium [9, 10], possibly close to northern Andøya, we hypothesised that lemmings sampled near this region would occupy a basal position in the phylogeny and exhibit relatively high genome-wide diversity.

## Results

### De novo assembly and annotation

We generated a de novo assembly for *Lemmus lemmus* (sample information: Additional file 1: Table S2), with a final size of 2.65 Gb. The total number of sequenced bases was 2,654,752,195, with an average coverage of 35 × . The scaffold assembly consisted of 170 scaffolds, and the contig assembly of 269 contigs. Furthermore, the mitochondrial genome was assembled. The scaffold N50 was 104 Mb, and the contig N50 was 51 Mb. Based on the mammalian database, the BUSCO completeness was 96.3%. Repetitive regions made up 45.1% of the genome (Tables 1 and 2).

**Table 1:** Summary of the BUSCO scores for the de novo assembly

Category	Score/percentage (Mammalia)	Score/percentage (Eukaryota)
Complete BUSCOs	8885/96.3%	254/99.6%
Complete and single-copy BUSCOs	8615/93.4%	230/90.2%
Complete and duplicated BUSCOs	270/2.9%	24/9.4%
Fragmented BUSCOs	64/0.7%	1/0.4%
Missing BUSCOs	277/3.0%	0/0.0%
Total BUSCO groups searched	9226/100%	255/100%

**Table 2:** Summary of the de novo assembly statistics

Category	Score
Number of scaffolds	170
Number of contigs	269
Total length	2.65 Gb
Percent gaps	0.001
Scaffold N50	104 Mb
Contigs N50	51 Mb

The final gene annotation resulted in the identification of 27,958 genes and 63,771 mRNA transcripts. BUSCO analysis demonstrated a high level of completeness, with 93.6% of BUSCOs classified as complete, including 1.7% duplicated. Additionally, 1.7% were found to be fragmented, and 4.7% were missing. Functional annotation assigned protein domains to 85.3% (24,403) of genes and 92.5% (59,000) of mRNAs, based on domain matches across multiple databases, including Pfam, CDD, and Gene3D. Furthermore, 79.5% of genes and 87.8% of mRNAs were assigned a gene name.

### Dataset

We resequenced 86 Norwegian lemming (*Lemmus lemmus*) whole genomes, with sample locations spanning across the species distribution (Fig. 1A), to an average coverage of between 2.5 × and 5 × . The resequenced genomes were analysed together with high-coverage genomes (15 × to 21 ×) obtained from a previous study by Lord et al. [11] (European Nucleotide Archive (ENA) study accession: PRJEB87511 [23]), from five *Lemmus lemmus* from the sites Borga, Kebnekaise and the Kola peninsula, two Western Siberian lemmings (*Lemmus sibiricus*) from the Kanin peninsula, one Eastern Siberian lemming (*Lemmus paulus;* synonymous with *Lemmus sibiricus* East*, Lemmus portenkoi, Lemmus bungei,* and *Lemmus ognevi*) from the Wrangel island, and one Nearctic brown lemming (*Lemmus trimucronatus*) from Alaska [11]. The samples were grouped based on geographic areas, mainly following the grouping used by Lagerholm et al. [6] (Fig. 1A): Kola peninsula, Varangerhalvøya, Sennalandet, Utsjoki, Pallasjärvi, Kittilä, Kilpisjärvi, Kebnekaise, SarPad, Vindelfjällen, Borga, Helags, Fulufjället, Åmotsdalen, Finse and Møsvatn. Here, SarPad refers to samples from Sarek and Padjelanta, Borga to samples from Børgefjell, Borgafjäll and Gussvattnet, Fulufjället comprises the samples from Fulufjället and Rendalen, and Åmotsdalen corresponds to samples from Åmotsdalen and Svånå. Pallasjärvi was grouped separately from Kittilä despite their close geographic proximity, based on the genetic differences identified previously [6]. An overview of the complete dataset is found in Additional file 1: Table S1. A kinship analysis performed using READv2 [24] (see Additional file 2: Fig. S1) revealed that IF085 and IF089 are first degree relatives.

### Population structure and phylogeny

In the PCA, Norwegian lemmings of the same or geographically close subregions form distinct genetic clusters (Fig. 1B), falling along a south-to-north gradient along PC1. PC2 explains less variation than PC1, and mainly separates the individuals from both the Kola Peninsula and Pallasjärvi, likely influenced by low genetic diversity. Notably, individuals from the Kola Peninsula form a genetically distinct cluster separated from the rest of Fennoscandia. Individuals from Pallasjärvi fall on a gradient along PC2. The only exception to this clear grouping is the samples from Kittilä, collected during a peak year. Two Kittilä samples fall close to the Pallasjärvi samples, while two samples cluster with the Utsjoki samples, and one sample falls with the Varangerhalvøya samples. The remaining three samples from Kittilä fall together, close to the individual from Sennalandet.

**Fig. 2:**
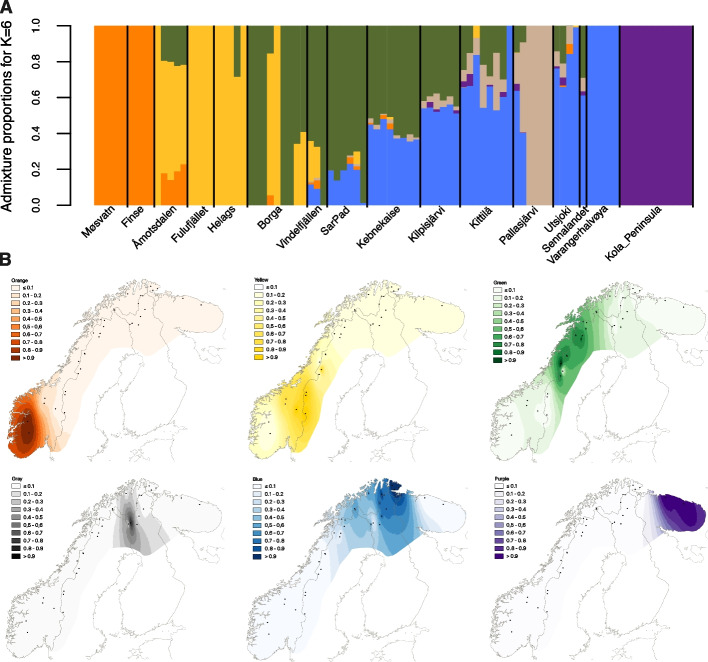
**A** Admixture proportions of the 91 Norwegian lemmings for *K* = 6, reflecting the clustering observed in the PCA. The individuals are sorted by sampling location, ordered from south to north. Colours indicate the ancestry proportion of the six components assigned for each individual. **B** The distribution of the six components is shown interpolated onto a map of Fennoscandia. Colours correspond to the ancestry components in the admixture plot, with the beige component being shown in black. Higher saturation of the colours indicates a higher presence of the component, following the legends next to each of the six maps

We tested the admixture proportions for *K* = 2 to *K* = 10 (Additional file 2: Fig. S2). *K* = 6 revealed the most similar results to the PCA, and lemmings from the same sampling locations show similar admixture proportions (Fig. 2A). Here, one component comprises the two southernmost subregions Møsvatn and Finse (in orange), while the second component (in yellow) is the main contributor to the southern subregions Åmotsdalen, Fulufjället, and Helags, and also found in some individuals from Borga and Vindelfjällen. The third and fourth components (in green and blue) are mainly found in the central to northern locations, with the third component (green) decreasing and the fourth component (blue) increasing towards the north. The Norwegian lemmings from Pallasjärvi show a distinct ancestry component (in beige), again indicating a genetic differentiation from all the other samples, despite the geographic proximity to Kittilä. The purple component is unique to the individuals from the Kola Peninsula, further supporting the genetic distinctness of the subregions. We interpolated the admixture plots onto a map of Fennoscandia (Fig. 2B), which resulted in a clear geographic distribution of the six genetic clusters.

**Fig. 3:**
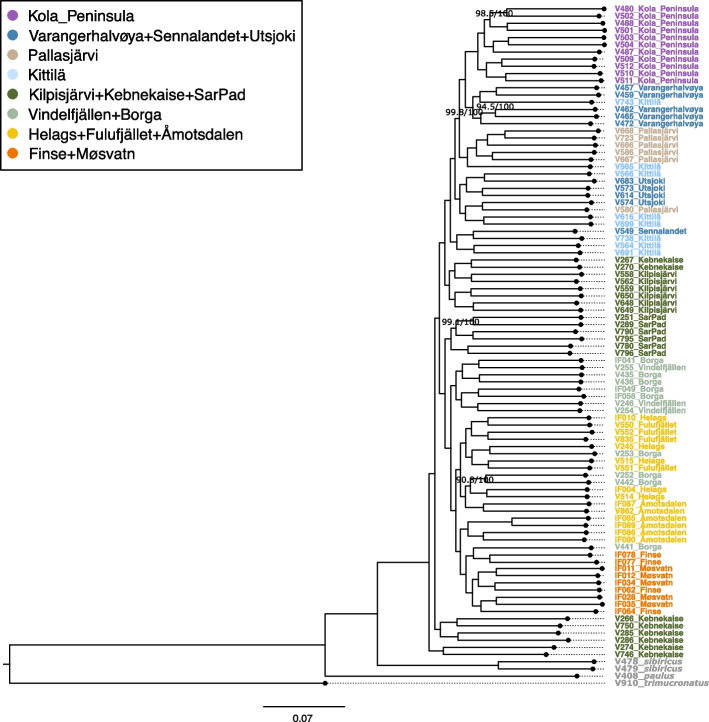
Autosomal maximum likelihood tree of the 95 lemmings, based on 5,215,512 sites and generated in IQ-TREE v2.3.5 [25]. The dataset comprises 91 *L. lemmus*, two *L. sibiricus*, one *L. paulus*, and one *L. trimucronatus* serving as outgroup (grey). Labels and colours indicate sample id and sample location. Branch labels correspond to the SH-aLRT support and the ultrafast bootstrap values. Nearly all nodes had 100/100 support, and only lower support values are being displayed. Distances were measured based on variable sites

We reconstructed an autosomal maximum likelihood tree (Fig. 3), using *L. trimucronatus* as an outgroup. All *L. lemmus* individuals form a monophyletic clade, as a sister species to *L. sibiricus*. The Norwegian lemmings cluster within two clades, each one comprising all samples north and south of Kebnekaise, respectively. Most samples from Kebnekaise fall basal of these two clades. Within the southern clade, the samples from Møsvatn and Finse in southernmost Norway cluster together with one sample from Borga (V441). Moreover, samples from the southern locations Åmotsdalen, Fulufjället and Helags mainly fall together, including a few samples from Borga. The remaining samples from Borga and all samples from Vindelfjällen form a third cluster. Within the northern clade, the Norwegian lemmings from Kilpisjärvi form a cluster with two additional samples from Kebnekaise. Similar to the PCA, the samples from Kittilä fall closely to samples from Pallasjärvi, Utsjoki, Sennalandet and Varangerhalvøya respectively. With the exception of V580, all Pallasjärvi samples form one cluster. Moreover, the Norwegian lemmings from Kola Peninsula form a monophyletic clade, consistent with the results of PCA and Admixture plots. A highly similar topology was also recovered in the Bio-NJ phylogenetic tree (Additional file 2: Fig. S3).

In contrast to the autosomal phylogeny, mitochondrial haplotype network (Additional file 2: Fig. S4) did not reveal such a clear structure. We find three major haplogroups, of which two contain samples from northern, central, and southern Fennoscandia. The third clade comprises all samples from the Kola peninsula together with samples from SarPad, Kebnekaise, Kilpisjärvi, Utsjoki, and Pallasjärvi.

### Genetic diversity

**Fig. 4:**
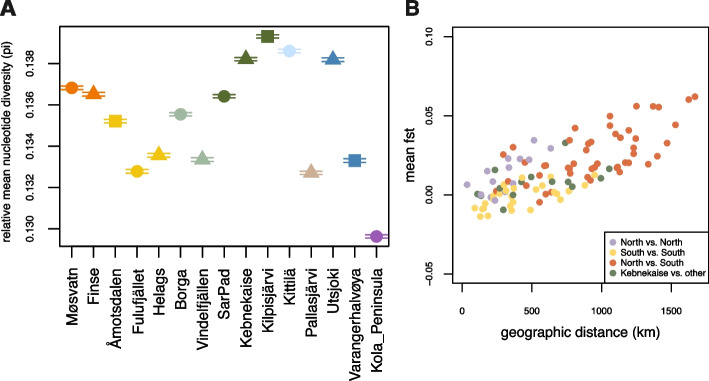
**A** Relative mean nucleotide diversity (π) of the Norwegian lemmings grouped by subregion, estimated based on variable sites in the dataset. Bars indicate the standard error. **B** Pairwise comparison of the mean Weir and Cockerham Fst estimate [26] of the Norwegian lemmings per subregion on the *y*-axis, with geographic distance in kilometres on the *x*-axis. Colours indicate the geographic locations of the subregions in the pairwise comparison, north and south of Kebnekaise respectively

Nucleotide diversity (π) was estimated to be the highest in Kilpisjärvi (0.13931) and lowest in the Kola Peninsula (0.12963) and Pallasjärvi (0.13271) (Fig. 4A). In a pairwise comparison of sampling locations, our results show that the genetic divergence between subregions, measured as mean Fst, increases with further geographic distance (Fig. 4B). Moreover, the mean Fst among Norwegian lemmings from southern subregions (yellow) is lower than among northern subregions (purple).

## Discussion

### De novo reference genome

In this study, we present a de novo reference genome assembly for the Norwegian lemming (*Lemmus lemmus*). The scaffold assembly consists of 170 scaffolds and the mitochondrial genome. The first 26 scaffolds are comparatively large, possibly corresponding to chromosomes. This matches the lemming karyotype, which is 2n = 50 [27], with 24 autosomes and two sex chromosomes. Our analyses suggest that scaffold 3 corresponds to the X chromosome, while scaffold 26 was identified as the Y chromosome (Additional file 1: Table S3).

### Population structure in the Norwegian lemming

Our findings suggest that the Norwegian lemming is structured in distinct subpopulations across the Fennoscandian alpine and arctic tundra. The relationship between the different subpopulations follows an isolation-by-distance pattern, where genetic differentiation between subregions increases with geographic distance (Fig. 4B). In the PCA (Fig. 1B), each subregion forms either a unique cluster or a cluster with the closest neighbouring subregions. The presence of these subpopulations across the species’ range is also apparent in the admixture plot (Fig. 2A) and the autosomal phylogeny (Fig. 3), which reveals clear genetic clusters from south to north. This pattern is consistent throughout the sample collection period from 1967 to 2022. Future additional sampling would be helpful to confirm the geographic delineation of the different clusters.

Previous research suggested that long-distance migrations during peak years can lead to gene flow and thus homogenisation of the gene pools [6, 7]. Our results are consistent with this hypothesis (Figs. 1B, 2A, and 4B); however, our findings indicate that this pattern is restricted to neighbouring subregions and is mainly present in the southern Scandes. The southern subregions appear to be more closely related to each other than the northern ones (Figs. 1B, 2A, and 4B), in line with previous findings [6]. For instance, we find that the gene pools of the southern regions Møsvatn and Finse (average distance ~ 90 km), and the gene pools of Åmotsdalen, Fulufjället, and Helags (average distance between ~ 145 and ~ 165 km) appear nearly fully homogeneous.

The higher genetic differentiation among northern subregions indicates a lower frequency of gene flow among, and/or higher genetic drift within, the subpopulations. For instance, we find genetic differentiation between the subpopulations of Varangerhalvøya and Utsjoki (Figs. 1B and 3), for which the average geographic distance is ~ 135 km. One reason could be the longer intervals (25–35 years) of high peak years and migrations of lemmings in northern Fennoscandia, and thus more limited dispersal of the Norwegian lemming [3, 4, 6]. Moreover, long low-density periods could also result in higher genetic drift within the northern subpopulations. However, the Kittilä samples, which were collected during a peak year, do not form a monophyletic cluster, further highlighting that recurrent but restricted gene flow continues to shape the population structure in northern Fennoscandia.

We note that the Norwegian lemmings from the Kola peninsula and Pallasjärvi form highly distinct clusters in both the PCA and Admixture analyses (Figs. 1B and 2A). Furthermore, relative mean nucleotide diversity was found to be the lowest among the Kola peninsula individuals, but also comparably low for the Pallasjärvi lemmings (Fig. 4A), possibly being one of the underlying factors influencing the unique clustering in the PCA. Interestingly, our results suggest no differentiation between the Kola Peninsula lemmings, despite ~ 150 km distance between the two sampling locations. The genetic uniqueness of these lemmings could be explained by the comparably large geographic distance (min. average distance ~ 330 km) of contiguous taiga forest between the Kola peninsula and the remaining subregions, resulting in a higher degree of differentiation of this subpopulation. Furthermore, a prolonged period without lemming peaks in the Kola Peninsula from 1987 to 2007 [28] has been reported, which may be indicative of a very small population size that eventually resulted in the genetic patterns observed.

The distinctiveness of Pallasjärvi is surprising given the close proximity to the other Kittilä sampling locations (~ 30 km), but consistent with previous findings based on microsatellite data [6]. The Pallasjärvi area comprises a set of isolated mountains in northern Finland that experienced comparatively high lemming abundance in 1970 and 1978 [4]. However, after the latter year, lemming presence was extremely low at Pallasjärvi: no lemmings were caught in the regular annual monitoring trappings, although sporadic signs indicated their presence. This lasted until spring 2010, when the first lemmings after 1978 were captured, during a very early phase of population increase, before any visible migratory movements connected with higher densities (Henttonen, pers. comm.). Based on this, we hypothesise that the genetic uniqueness of the Pallasjärvi population as well as the comparably low nucleotide diversity in this isolated mountain area (Fig. 1B) are consequences of genetic drift during its > 30 year isolation at low population size. In cyclic populations, we would expect low-abundance periods to reduce genetic diversity within subpopulations, whereas peak years with increased dispersal and migration can promote gene flow between subpopulations, and thereby lead to increased diversity.

### Genome-wide patterns and postglacial history

Previous research based on short mitochondrial markers suggested that a small population of the Norwegian lemming survived the Last Glacial Maximum in a local northern refugium and subsequently recolonised Fennoscandia [9, 10]. One possible location for such a refugium could have been the northernmost tip of the Norwegian island Andøya, which has been reported as ice-free during the LGM [10, 17, 29]. Generally, our findings are consistent with the previously hypothesised glacial survival in the Andøya region. In the phylogenetic tree based on nuclear genomes, we observe a basal placement of the Kebnekaise samples (Fig. 3), which are the samples in our dataset that are geographically closest to Andøya. The BioNJ tree (Additional file 2: Fig. S3) revealed a similar topology, with a northern and a southern clade and the Kebnekaise lemmings falling basal of each clade. In contrast to the distinct population structure in the autosomes, the mitogenomes did not reveal any clear phylogeographic patterns (Additional file 2: Fig. S4). The lack of structure in the mitogenomes, together with a clear structure in the autosomal data, is consistent with expectations under a scenario of rapid postglacial expansion from a single refugial origin, with subsequent genetic drift and isolation of subregions during the Holocene. Moreover, we find a high nucleotide diversity in Kebnekaise and Kilpisjärvi (Fig. 4A), which would be expected for subpopulations close to where a postglacial expansion originated.

It is important to note, however, that since our analyses are based on modern genomes alone, we have only limited power to discriminate between a scenario of local survival versus a uni-directional, postglacial recolonisation from a glacial refugium outside of Scandinavia. The postglacial recolonisation history of *Lemmus* sp. could be further investigated using ancient genome-wide data, both from within and outside Fennoscandia. This is particularly the case for cold adapted species whose distributions have contracted during the Holocene, since an ancient DNA approach is necessary to recover genome-wide data from potential source populations that became extinct at the end of the Pleistocene. Moreover, ancient whole-genome data could be used to build on the work of Lord et al. 2025 [11] to further investigate the evolutionary history of the Norwegian lemming and its sister species, as well as to explore when and where the unique genetic adaptations of the species have evolved in real time.

## Conclusions

The high-quality reference genome presented here will likely constitute a valuable resource for other studies, such as those on environmental DNA, as well as future research on the evolutionary genomics of the Norwegian lemming. Leveraging this reference genome, we here generated a dataset of 86 resequenced genomes sampled from across Fennoscandia. We revealed the presence of geographically distinct subpopulations and an isolation-by-distance pattern among Norwegian lemming subpopulations. Furthermore, our results are consistent with the previously suggested hypothesis that the species originates from a small population that survived the Last Glacial Maximum in a local northern refugium, and subsequently expanded across Fennoscandia.

## Methods

### De novo assembly and annotation

A Norwegian lemming sample (lab ID: JM03) from Helags (County Jämtland, coordinates: 62.914889, 12.502212) was used to generate a de novo genome assembly (Additional file 1: Table S2).

### DNA and RNA extraction for de novo assembly

DNA and RNA extraction of sample JM03 was performed at the National Genomics Infrastructure (NGI) Uppsala (Uppsala Genome Center). High molecular weight (HMW) DNA from ~ 10 mg of spleen was extracted using the Monarch HMW DNA Extraction Kit for Tissue (NEB, #T3060S/L) following the protocol “High Molecular Weight DNA Extraction from Tissue” described in the instruction manual (Version 1.0_10/20). The tissue was cut into the smallest possible pieces prior to using a pestle homogeniser. DNA was eluted in 200 µl kit EB and left at room temperature with gentle mixing at 100 rpm on a platform rocker for several days to increase homogeneity before performing QC. Afterwards the extracted DNA was stored at 4 °C until processed further. RNA was extracted from *Lemmus lemmus* muscle and brain tissue using the TRIzol Reagent and Phasemaker Tubes Complete System (Invitrogen Cat #A33250) following the Invitrogen user guide (Pub. No. MAN0016163 Rev. A.0) except for steps 2.a. and 3.d., which were omitted. RNA was resuspended in 87.5 µl RNase-free water and immediately subjected to DNase treatment followed by purification according to the RNeasy Micro Handbook (pages 74 and 53).

### Iso-Seq sequencing for de novo assembly

One hundred fifty nanograms of RNA extracted from muscle was combined with 150 ng of RNA extracted from brain tissue. From a total of 300 ng of RNA one PacBio SMRTbell™ library was prepared as described in “Procedure & Checklist – Iso-Seq™ Express Template Preparation for Sequel® and Sequel II Systems” (PN 101–763–800 Version 02 (October 2019)) using the NEBNext® Single Cell/Low Input cDNA Synthesis & Amplification Module, the Iso-Seq Express Oligo Kit, ProNex beads (Promega) and the SMRTbell Express Template Prep Kit 2.0. Sequencing was performed at NGI Uppsala (Uppsala Genome Center). One Sequel™ SMRT® Cell 8 M v3 was sequenced on Sequel IIe System using Sequel® II Sequencing Plate 2.0. The on-Plate Loading Concentration was 90 pM. Movie time was 24 h with a pre-extension time of 2 h.

### De novo sequencing

Input QC of the DNA was performed using Dropsense, Qubit and Femto pulse to evaluate concentration, purity and size. The sample libraries were prepared according to Pacbio’s Procedure & Checklist – Preparing HiFi SMRTbell® Libraries using the SMRTbell Express Template Prep Kit 2.0, PN 101–853-100 Version 05 (August 2021) using the SMRTbell Express Template Prep Kit 2.0. The samples were sheared on Megaruptor 3 with speed setting 31. An Ampure bead purification was performed after the shearing. The samples were size selected using SageElf, according to Pacbio’s protocol. Fractions 1–3 were used for sequencing. Quality control of sheared DNA and SMRTbell libraries was performed on Fragment analyser, using the Large Fragment standard sensitivity 492 kit. Primer annealing and polymerase binding were performed using the Sequel II binding kit 2.2. Sequencing was performed at NGI Uppsala (Uppsala Genome Center). Three Sequel™ SMRT® Cell 8 M v3 were sequenced on Sequel IIe System using the Sequel® II Sequencing Plate 2.0. Movie time was 30 h with a pre-extension time of 2 h.

At the NGI in Stockholm, Sweden, two Dovetail OmniC sequencing libraries were prepared following the instructions provided by the manufacturer (“Mammalian Samples Protocol version 1.3”, method “B”). The starting material to each prep was 10 mg of fresh-frozen liver tissue which had been ground to a fine powder. The finished OmniC libraries were pooled to fill 1/4th the capacity of a lane in an Illumina NovaSeq 6000 S4-300 v1.5 flowcell, then eventually sequenced in one using a 2 × 151 nucleotide (nt) setup.

### Genome assembly

PacBio HiFi reads were assembled into contigs using Hifiasm v0.16.0 with the default parameters [30]. Duplicates were removed by using purge_dups v1.2.6 [31]. The purged assembly completeness and false duplication rate were assessed by Merkuryfk v1.1, and BUSCO v5.4.3 [32] was run with the datasets eukaryota_odb10 and mammalia_odb10. The mitochondrial genome was detected by mitohifi v2.2 [33] and removed from the contig assembly. De novo scaffolding of the genome assembly was performed by first mapping the OmniC data to the contig assembly using BWA-MEM v0.7.17 [34] with options “−5SP -T0” then filtering using pairtools v0.3.0 [35] commands “parse”, “sort”, “dedup” and “split”. The draft scaffolds were produced by yahs v@42b8421 [36] and were manually curated in Juicebox (v. 2) [37].

### Genome annotation

Genome annotation relies heavily on high-quality evidence data. In this study, proteins were obtained from the UniProt Swiss-Prot database (568,363 proteins) [38]. RNA-seq data of brain and muscle from Illumina RNA-seq sequencing were assembled using fastp v0.23.2 [39], HISAT2 v2.1.0 [40], and StringTie v2.2.1 [41], following a Nextflow v22.10.1 [42] in-house pipeline [43]. Additionally, an Iso-Seq library comprising 238,050 full-length cDNAs generated using PacBio SMRT sequencing technology was used.

A species-specific repeat library was constructed using the RepeatModeler package v2.0.2a [44] to eliminate nucleotide motifs from low-complexity coding sequences. Repeat sequences were identified using RepeatMasker v4.1.2_p1 [45] and RepeatRunner (https://github.com/Yandell-Lab/RepeatRunner) to analyse highly divergent repeats and retro-element coding regions.

Gene builds were computed using the MAKER pipeline v3.01.02 [46]. The gene annotation process consisted of two primary steps: (1) an evidence-based build, where transcript alignments and reference proteins were used to generate consensus gene structures, and (2) an ab initio build, which leveraged evidence alignments along with a curated ab initio profile. Augustus [47] was trained for ab initio gene prediction using an in-house pipeline [43]. Functional annotation of genes and transcripts was performed using the translated CDS features of each coding transcript. BLAST v2.9.0 [48] and InterProScan v5.59–91.0 [49] were used to infer canonical protein names and functional predictions, with the results parsed and reconciled into a final annotation using an in-house pipeline [43].

The final annotation was evaluated using an in-house Perl script [50]. To improve the quality of gene models, evidence-based and ab-initio annotations are usually combined, using one as the base and supplementing it with the other for loci missing in the base set. In our case, the evidence-based annotation outperformed the ab initio run, which exhibited more duplicated genes, fewer mRNAs, and fewer mRNAs with both UTRs. Consequently, the evidence-based annotation was selected as the foundation and supplemented with ab initio predictions.

We also observed an unusually high number of single-exon genes lacking functional annotation, likely due to overprediction by MAKER. To improve annotation quality, single-exon genes without functional evidence (i.e. lacking domain or gene name) were removed, provided their removal did not impact BUSCO scores [51].

### DNA extraction, library preparation, DNA sequencing of population genomics data

Eighty-six modern Norwegian lemming (*Lemmus lemmus*) samples were collected between 1967 and 2022 across the present-day range of the species in Fennoscandia (Additional file 1: Table S1; Fig. 1A). DNA of the modern samples was extracted from a small piece of tissue using the DNeasy blood and tissue kit from Qiagen (Qiagen, Hilden, Germany) following the manufacturer’s protocol. Additionally, DNA extracts were obtained from a previous research project [6], in which a GeneMole MG10-000 robot and the MoleStrips DNA Tissue kit (Mole Genetics) were used. The TruSeq Nano DNA library preparation kit (Illumina Inc.) was used for DNA library building with 350 base-pair (bp) insert size, and sequencing was then performed on two NovaSeq 6000 S4 lanes with a 2 × 150 bp paired-end setup, at NGI, Uppsala.

### Mapping and BAM file processing

Sequence data processing was performed using the GenErode pipeline v0.5.1 [52]. The adapter trimming was run on the raw FASTQ files using fastp v0.22.0 [39], and a minimum read length of 30 was applied. Subsequently, the reads were mapped to the here presented *L. lemmus* reference genome, with the nuclear and mitochondrial DNA scaffolds concatenated, using BWA v0.7.17 mem algorithm [34]. Indels were realigned using GATK3 v3.7 [53]. Duplicates were identified and removed using Picard/3.1.1 MarkDuplicates (https://broadinstitute.github.io/picard/). The BAM files were filtered for a minimum mapping quality of 25 and minimum base quality of 30. Finally, BAM files were subsampled to an average coverage of 3 × using samtools v1.20 [54]. Additionally, genomic data from five previously published *L. lemmus*, two Western Siberian lemmings (*L. sibiricus*), one Eastern Siberian lemming (*L. paulus*), and one Nearctic brown lemming (*L. trimucronatus*) [11, 23] were included and processed as described above. De novo identification and masking of repetitive regions in the reference genome was performed with RepeatModeler v2.0.1 [44] and RepeatMasker v4.0.9 [45] as implemented in GenErode v0.5.1.

### Sex chromosome identification

Sex chromosome identification of the newly assembled genome was performed with a depth-based approach leveraging the known sex of previously published individuals [11]. For each scaffold, the depth of every position was obtained with SAMtools (including zero depth positions with the command: samtools depth -a) and then averaged. The profiles of average depth for scaffolds between males and females were confronted to identify X and Y scaffolds. In females, X chromosome scaffolds should have a depth comparable to the autosomes while Y chromosome ones should be ~ 0; whereas in males, X chromosome and Y scaffolds should have a depth that is ~ ½ of the autosomes. We identified scaffold 3 to be linked with the X chromosome, as well as potentially scaffold 63 and 167. The final size of scaffold 3 was 153 Mb, which corresponds to ~ 5.8% of the size of the assembly. Scaffolds 27, 28, 34, 68 and 88 were linked with the Y chromosome. Scaffold 26 shows a peculiar pattern having a depth of ~ 0 in females, but one that is comparable to autosomes in males. This could be the result of the large number of rodent-specific repeated sequences on the vast majority of this chromosome, as it occurs on the mouse Y chromosome [55]. The final size of the scaffolds linked with the Y chromosome, excluding scaffold 26, was 17 Mb, making up ~ 0.6% of the assembly. For further results see Additional file 1: Table S3.

### Relatedness

We estimated relatedness among the 91 Norwegian lemmings using READv2 [24]. Results are reported in the Additional file 2.

### Genotype calling and likelihoods

Genotype likelihood estimates and genotype calling were performed using ANGSD v0.940 [56], with the option -GL 1, filters for mapping quality of 25 and base quality of 30 (-minMapQ 25, -minQ 30), removing low quality and duplicated reads (-remove_bads 1), keeping uniquely mapped reads only (-uniqueOnly 1), keeping only reads for which both mates are mapped correctly (-only_proper_pairs 1), adjusting mapping quality for excess of mismatches (-C 50), with BAQ computation (-baq 1) [57], only keeping sites with a p-value below 1e-6 (-SNP_pval 1e-6), writing major minor alleles (-dogeno 1), inferring major and minor alleles from genotype likelihoods (-doMajorMinor 1), assuming known major and minor alleles (-doMaf 1), estimating the posterior genotype probability based on the allele frequency (-dopost 1), allowing for 5% missingness per site, and with repeats being masked. We obtained beagle and BCF files, containing 2,452,819 sites for only the 91 *L. lemmus* dataset used for PCA and Admixture plots, and 5,215,512 sites for all 95 *Lemmus* samples, used as input for all other analyses. The BCF files were converted to VCF files with BCFtools version 1.20 [58].

### PCA and admixture

A principal component analysis (Fig. 1B) of the Norwegian lemmings was performed using PCAngsd v1.11 [59], with beagle files as input and applying a minor allele frequency filter of 0.05. 2,444,131 sites were maintained after filtering. Scaffolds associated with sex chromosomes (3, 26, 27, 28, 34, 63, 68, 88, 167; Additional file 1: Table S3) and the mitochondrial scaffold were excluded. Moreover, sample IF089 was excluded due to relatedness with sample IF085. Admixture plots (Fig. 2) based on the genotype likelihoods of autosomal scaffolds were generated using NgsAdmix v32 [60], including a minor allele frequency filter of 0.05 and excluding sample IF089, using 2,444,100 sites. We tested with *K* = 2 to *K* = 10 (see Additional file 2: Fig. S2). Plotting was done using R v4.3.2 (2023–10–31) [18]. The data were interpolated onto a map of Fennoscandia using QGIS v3.40 with the Inverse Distance Weighted (IDW) interpolation method [61].

### Population differentiation (Fst) and relative nucleotide diversity (π)

We estimated the mean relative nucleotide diversity (π) per site for each sampling location using VCFtools v0.1.16 [62] (–sites-pi), with the VCF files of the autosomal scaffolds as input, which was then summarised as mean value per sampling location. Given that only sites that showed variation from the reference genome are included in the VCF file, the obtained values are primarily informative for the relative amount of genetic diversity when comparing the *Lemmus lemmus* subpopulations. Furthermore, we calculated the Weir and Cockerham Fst estimate [26] for each pair of sample locations using VCFtools v0.1.16 (–weir-fst-pop), and genotype likelihoods of the autosomal scaffolds as input. For both analyses, we grouped the *L. lemmus* samples by location and excluded IF089 due to relatedness with IF085. Moreover, Sennalandet (*n* = 1) was excluded from the analyses due to low sample size. Plots were created using R v4.3.2 (2023–10–31) [18]. Underlying data is found in Additional file 1: Table S4.

### Autosomal phylogeny

First, we converted the VCF files of autosomal scaffolds to MAP/PED format, with PLINK v1.9 [63], choosing a random allele when two different ones were present. We later converted the MAP/PED files into FASTA format using the ped2fasta script (https://github.com/gungorbudak/ped2fasta). Then we reconstructed a maximum likelihood tree based on 5,215,512 sites using IQ-TREE v2.3.5 [25], with testing for the best model (-m TEST; TVM + F + I + G4 was chosen as the best fitting model according to BIC), an ultrafast bootstrap approach [64] and SH-aLRT branch test [65] with 1000 replicates each (-B 1000; -alrt 1000). The phylogenetic tree was visualised using Figtree v1.4.4 (https://github.com/rambaut/figtree/releases/tag/v1.4.4). The Nearctic brown lemming (*L. trimucronatus*) was used as the outgroup.

Furthermore, we constructed a BioNJ tree (Additional file 2: Fig. S3) based on a whole genome identity-by-descent (IBS) matrix for the 95 lemming genomes. We used ANGSD v0.940 [56] to generate the IBS matrix, with the following settings: -doIBS 1 (random based sampling), -doCounts 1 (count base each position), -makeMatrix 1 (output an IBS matrix), -minInd 90 (maximum of 5% missingness), -uniqueOnly 1 (uniquely mapped reads only), -remove_bads 1 (removing reads of bad quality), -minMapQ 25 (mapping quality), -minQ 30 (base quality), -doMajorMinor 1 (inferring major and minor allele from genotype likelihoods), -GL 1 (samtools method for genotype likelihoods), -only_proper_pairs 1 (only reads with both mates properly mapped), with the repeat-masked bed file for autosomal scaffolds used as the sites file. The phylogeny was FastME2 [66], using the BioNJ tree building method with tree refinement using SPR. *L. trimucronatus* was used as the outgroup and visualisation was done in Figtree v1.4.4 (https://github.com/rambaut/figtree/releases/tag/v1.4.4).

### Mitochondrial haplotype network

Mitochondrial fasta files were generated using ANGSD v0.940 [56] doFasta 2 (most common base), with a minimum base quality filter of 30 (-minQ 30) and a mapping quality filter of 25 (-minmapQ 25), a minimum depth of 10 × per site (-setMinDepth 10), removing duplicates, non-primary and failed reads (-remove_bads 1), as well as reads with more than one best hit (-uniqueOnly 1). The consensus mitogenome sequences had a length of 16,335 bp. A median-joining mitochondrial haplotype network of the 91 *Lemmus lemmus* samples was generated using popART [67, 68] using default settings with epsilon = 0.

## Supplementary Information

Additional file 1. Table S1: Sample information. Table S2: Sample information for the de novo genome assembly. Table S3: Sex-chromosome identification. Table S4: Underlying data for Fig. 4.

Additional file 2. Fig. S1: Kinship estimates from READv2. Fig. S2: Admixture proportions of the Norwegian lemming samples, with K=2 to K=10. Fig. S3: Autosomal BioNJ phylogeny based on an ibs-matrix and generated using FastME2. Fig. S4: Mitochondrial haplotype network generated in popART.

## Data Availability

The de novo reference genome assembly is available at European Nucleotide Archive (ENA) under the study number PRJEB68357. The raw read sequencing data of the 86 Norwegian lemming genomes is deposited on the ENA under the project number PRJEB98905 (sample accession numbers: ERS28343745 - ERS28343830).

## Abbreviations

bp: Base pair
ENA: European Nucleotide Archive
HMW: High molecular weight
LGM: Last Glacial Maximum
*L.*: *Lemmus*
NAISS: National Academic Infrastructure for Supercomputing in Sweden
NGI: National Genomics Infrastructure Sweden
nt: Nucleotide
SciLifeLab: Science for Life Laboratory
UPPMAX: Uppsala Multidisciplinary Centre for advanced computational Science
