# Supplementary material for: Whole genomes reveal subpopulations and isolation-by-distance patterns in the Norwegian lemming
Source: BMC Biol. 2026 Mar 6;24:93. doi: 10.1186/s12915-026-02568-w (PMC13064042; doi:10.1186/s12915-026-02568-w)
Supplement: Supplementary file 2 — Additional file 2. Fig. S1: Kinship estimates from READv2. Fig. S2: Admixture proportions of the Norwegian lemming samples, with K=2 to K=10. Fig. S3: Autosomal BioNJ phylogeny based on an ibs-matrix and generated using FastME2. Fig. S4: Mitochondrial haplotype network generated in popART. [file 12915_2026_2568_MOESM2_ESM.pdf]

Additional file 2

Whole genomes reveal subpopulations and isolation-by-distance patterns in the Norwegian lemming

Isabelle Sofie Feinauer, Francesco Ravasini, Vendela Kempe Lagerholm, Johannes Måsviken, Remi-Andre Olsen, Lucile Soler, Estelle Proux-Wera, Ignas Bunikis, Henrik Lantz, Kerstin Lindblad-Toh, Dorothee Ehrich, Rolf A. Ims, Heikki Henttonen, Nina E. Eide, Øystein Flagstad, Karin Norén, Anders Angerbjörn, Love Dalén

Relatedness

Kinship analysis of the 91 Norwegian lemmings was performed using READv2. Results revealed IF085 and IF089 to be first-degree relatives. Both samples were collected in Oppdal in 2013. No other closely related pairs were detected in the dataset. To avoid biases, IF089 was excluded from PCA, Admixture, and estimation of nucleotide diversity and Fst.

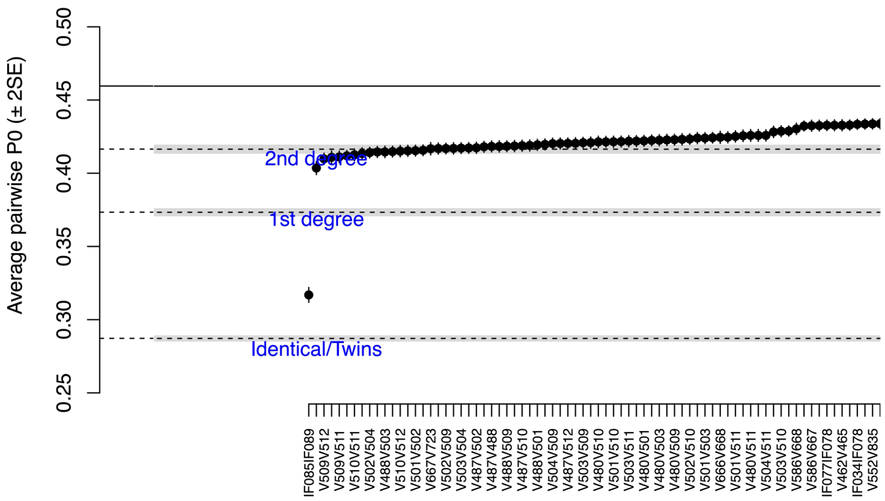

Fig. S1: Kinship estimates from READv2, with IF085 and IF089 identified as first degree relatives. The figure has been shortened for readability removing the majority of unrelated pairs on the right end of the plot.

Admixture

Admixture graphs were generated in NgsAdmix v32 from K=2 to K=10, with filtering for minor allele frequency of 0.05 and excluding sample IF089.

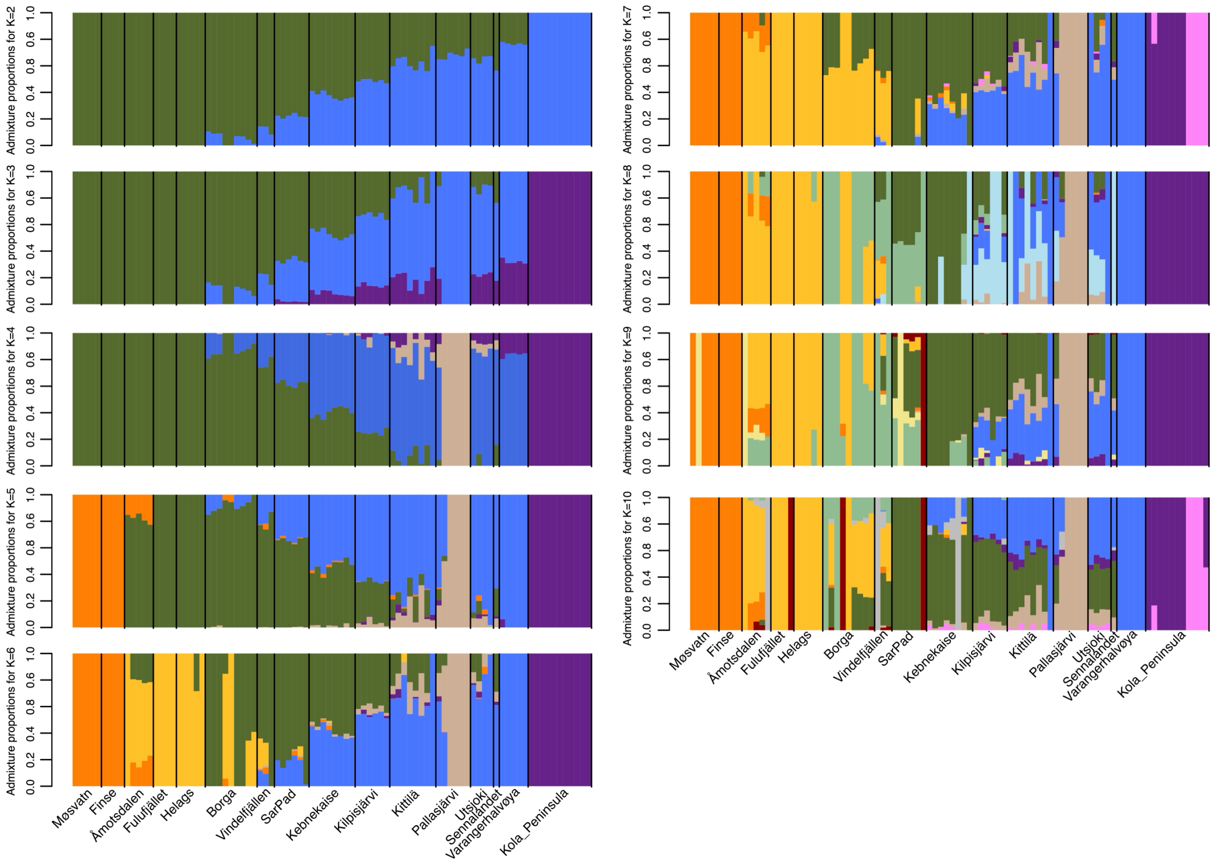

Fig. S2: Admixture proportions of the Norwegian lemming samples, with K=2 to K=10.

BioNJ phylogeny

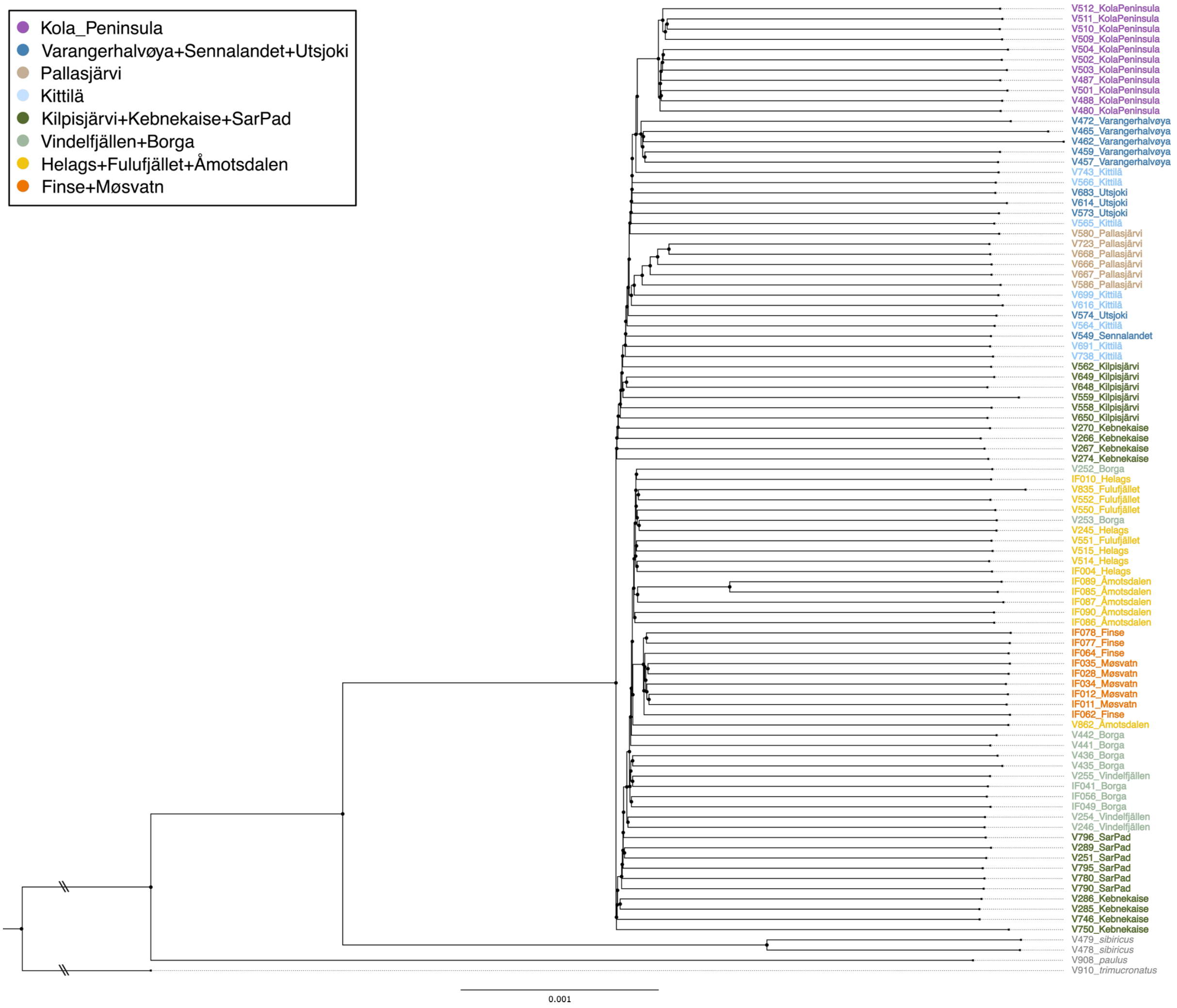

Fig. S3: Autosomal BioNJ phylogeny of the 95 lemmings, based on an ibs-matrix and generated using FastME2.

Mitochondrial haplotype network

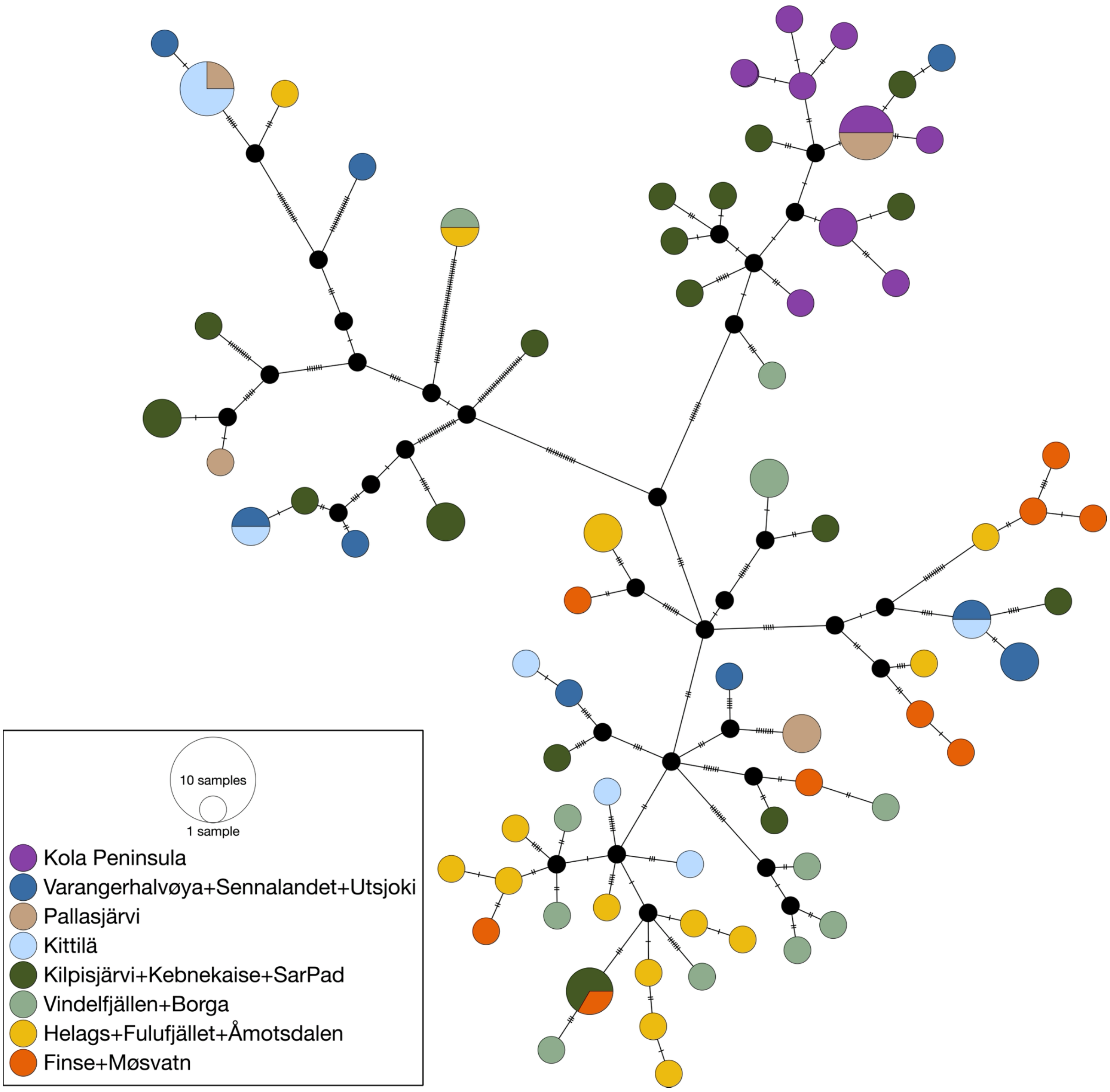

Fig. S4: Mitochondrial haplotype network of the 91 Norwegian lemming samples, generated in popART.
